# Application of NMR and Chemometric Analyses to Better Understand the Quality Attributes in pH and Thermally Degraded Monoclonal Antibodies

**DOI:** 10.1007/s11095-023-03600-2

**Published:** 2023-10-05

**Authors:** Victor A. Beaumont, Lucy Liu, Heliang Shi, Jason C. Rouse, Hai-Young Kim

**Affiliations:** 1 Pfizer, Inc. BioTherapeutics Pharmaceutical Sciences, Analytical Research and Development, 1 Burtt Road, Andover, MA 01810 USA; 2 Present Address: Pfizer, Inc. Pharmaceutical Sciences Small Molecules, Analytical Research and Development, Discovery Park, Ramsgate Road, Sandwich, CT13 9FF UK; 3 Pfizer, Inc. Global Product Development, Oncology & Rare Disease Statistics, New York City, NY 10001 USA

**Keywords:** antibodies, biopharmaceutical characterization, chemometrics, nuclear magnetic resonance (NMR) spectroscopy, protein structure

## Abstract

**Purpose:**

Nuclear magnetic resonance (NMR) spectroscopy provides the sensitivity and specificity to probe the higher order structure (HOS) of monoclonal antibodies (mAbs) for potential changes. This study demonstrates an application of chemometric tools to measure differences in the NMR spectra of mAbs after forced degradation relative to the respective unstressed starting materials.

**Methods:**

Samples of adalimumab (Humira, ADL-REF) and trastuzumab (Herceptin, TRA-REF) were incubated in three buffer-pH conditions at 40°C for 4 weeks to compare to a control sample that was left unstressed. Replicate 1D ^1^H and 2D ^1^H-^13^C HMQC NMR spectra were collected on all samples. Chemometric analyses such as Easy Comparability of HOS (ECHOS), PROtein FIngerprinting by Lineshape Enhancement (PROFILE), and Principal Component Analysis (PCA) were applied to capture and quantitate differences between the spectra.

**Results:**

Visual and statistical inspection of the 2D ^1^H-^13^C HMQC spectra of adalimumab and trastuzumab after forced degradation conditions shows no changes in the spectra relative to the unstressed material. Chemometric analysis of the 1D ^1^H NMR spectra shows only minor changes in the spectra of adalimumab after forced degradation, but significant differences in trastuzumab.

**Conclusion:**

The chemometric analyses support the lack of statistical differences in the structure of pH-thermal stressed adalimumab, however, it reveals conformational changes or chemical modifications in trastuzumab after forced degradation. Application of chemometrics in comparative NMR studies enables HOS characterization and showcases the sensitivity and specificity in detecting differences in the spectra of mAbs after pH-thermal forced degradation with respect to local and global protein structure.

**Supplementary Information:**

The online version contains supplementary material available at 10.1007/s11095-023-03600-2.

## Introduction

Most approved biologics are therapeutic monoclonal antibodies (mAbs) and they are increasingly occupying a larger share of the pharmaceutical market because of their proven efficacy and safety against a vast range of diseases [1–6]. The connection between mAb biological activity and its inherent higher order structure (HOS) is widely understood and appreciated, such that changes in structure can lead to a gain or loss of function in the biotherapeutic. Aggregation, fragmentation, and potential chemical modifications (i.e., deamidation, isomerization, and oxidation) are typically investigated by both longer-term, real-time stability studies with the drug product presentation (i.e., vial, syringe, etc.) and accelerated forced degradation studies using pH extremes, chemical oxidation, light exposure, and elevated temperatures. Identifying sites and regions of instability or vulnerability and their potential effect on potency is crucial to understanding the various mAb structure–function relationships.

The HOS of therapeutic mAbs is assessed with multiple biophysical techniques during process and product development to ensure that structural integrity, product quality, safety, and efficacy are maintained in the manufactured drug substance from batch to batch. There are several mainstream biophysical techniques that can be used to monitor HOS during biotherapeutic development such as far-UV and near-UV circular dichroism (CD), Fourier transform infrared (FTIR) spectroscopy, fluorescence spectroscopy, and differential scanning calorimetry (DSC) [7–12]. Cryogenic electron microscopy (cryo-EM), X-ray crystallography, nuclear magnetic resonance (NMR) spectroscopy, hydrogen/deuterium exchange mass spectrometry (HDX/MS), and fast photochemical oxidation of proteins-mass spectrometry (FPOP-MS) are higher resolution techniques that currently require subject matter experts to acquire, process, and interpret the data generated in a biopharmaceutical lab [13–18]. Among these methods, nuclear magnetic resonance (NMR) spectroscopy is an emerging technique to characterize HOS either as an orthogonal technique to provide further evidence towards structural change or as a complementary technique to address deficiencies in the structural characterization from other biophysical techniques [17]. NMR is highly sensitive to changes in the chemical environment of observable nuclei and provides a wide range of structural resolution from chemical modifications and local conformational changes to global-level HOS changes. Lastly, NMR affords native-state, in-solution protein HOS characterization in a variety of formulation buffers, which allows for structural characterization analyses unadulterated by extensive sample manipulation.

Recent advancements in NMR methods and hardware overcome the challenges associated with high molecular mass proteins such as mAbs. Such challenges include greater line broadening as the rotational correlation time decreases, the lack of isotopic enrichment schemes for nuclei with low natural abundance, and the unfavorable gyromagnetic ratios of heteronuclei (^13^C or ^15^N). While ultra-high field NMR instruments (≥ 900 MHz) are now commercially available and inherently provide improved sensitivity and resolution for state-of-the-art protein characterization, they are too costly for most laboratories. At this time, NMR magnetic field strengths of 500–800 MHz are the predominant instrument configuration for most biopharmaceutical applications and laboratories [19, 20]. Furthermore, cryogenically cooled NMR probes (cryoprobes or cold probes) are now commonplace and enhance sensitivity by reducing the thermal noise generated by the electronics of the instrument [21]. Additionally, researchers have developed pulse sequences and acquisition methods for high-quality spectra of intact mAbs by improving the sensitivity and resolution [22–24]. The spectra of mAbs are greatly improved by suppressing both solvent signals and small molecule excipient peaks by using either diffusion filters or novel selective excitation techniques [25–27]. In the context of 2D NMR, sensitivity and resolution are limited by the number of scans and t_1_ time domain points acquired, which often lead to long experiment times (hours to days) for large molecules of natural isotopic abundance. Recent solutions to this problem come in the form of non-uniform sampling and rapid acquisition methods (i.e., selective optimized flip angle short transient or SOFAST pulse schemes) that greatly reduce experiment times [28, 29]. Additionally, the use of ^1^H-^13^C methyl 2D NMR spectroscopy improves sensitivity and line shape while still informing on changes to the global structure because methyl groups are well dispersed throughout mAb structures [24, 30–34].

Although employing the innovations above greatly improve the spectra of mAbs at natural isotopic abundance, full-length resonance assignments are still impractical given the large number of resonances, extensive signal overlapping, and exchange broadening. Combined chemical shift differences (CCSD) have been used for structural analyses, but are best implemented when peaks are well resolved and assigned [35–37]. However, this analytical method is tedious and unfit for large molecules because peak picking becomes laborious, peaks are not easily assigned, and poor resolution can influence the quality of the result. Rather, structural characterizations are performed by fingerprinting methods where the totality of the spectra is considered unique to the structure of the molecule. Analyses of the fingerprint of the spectrum simplify the structure characterization while still extracting HOS information because the NMR is sensitive to the chemical environment and structure of the protein. Several labs across all sectors have also developed and incorporated chemometric and fingerprinting methods for faster analysis and quantitative metrics for assessing HOS and measuring differences between NMR spectra of related materials [17, 27, 30, 32, 33, 38–48].

Three such chemometric tools include protein fingerprinting by line shape enhancement (PROFILE), principal component analysis (PCA), and easy comparability of HOS (ECHOS) analysis. PROFILE uses the excipient-suppressed ^1^H 1D spectra and generates a fingerprint spectrum by subtracting a low-resolution contour of the spectrum from the original [27]. A Pearson value (R value) or similarity score is then calculated between two spectra to assess the differences. PCA is a well-documented dimensionality-reduction technique, but in the context of 1D or 2D NMR it involves binning the entire spectrum and linearizing it to form a row vector of intensities at specific bins [32, 49]. This is repeated for all spectra in a library such that each row defines a spectrum, and each column defines a bin in the matrix, to which the PCA algorithm is then applied. The distances between the resulting scores or clusters are then used to assess differences between spectra. The ECHOS analysis is an intensity cross-correlation between two 2D spectra where a linear regression can be determined [30]. The correlation coefficient or R value of the linear regression is used to assess the difference between spectra. A commercial package (MBioHOS by MestreLab Research, https://mestrelab.com/software/mnova/mnova-biohos/) is available for nonexperts of NMR or statistical analysis to facilitate data processing and chemometric analysis of NMR spectra using these tools. However, it is important to note that there are no universally established acceptance criteria or benchmark metrics for these chemometric methods, and the interpretation of the results from these methods are dependent on the study or project [47].

In this work, we demonstrated that the combination of NMR and chemometrics afford the sensitivity and specificity to detect and quantitate changes in the protein structure of pH-thermally stressed mAbs. As a case study, we used two different IgG1 mAbs (Humira, ADL-REF, adalimumab and Herceptin, TRA-REF, trastuzumab) that behave differently to forced degradation conditions involving elevated temperature (40°C) and selected buffer-pH conditions (pH 4.5, 5.8, and 7.5). Previous liquid-chromatography-mass spectrometry (LC–MS) low-artifact peptide mapping data has shown that while adalimumab does not have significant modifications (< 1%) in the complementarity determining regions (CDRs) after being subjected to 40°C at various pH conditions for four weeks, the CDRs of trastuzumab did have significant Asn deamidation (11–72%) and Asp isomerization (37–43%) [50]. Relative potency results (data not shown) of adalimumab and trastuzumab at elevated temperatures for a month in the formulation buffer under the same conditions show that the degree of antigen binding in trastuzumab significantly decreases, while adalimumab retains full binding activity. This implies that the protein modifications in the CDRs of trastuzumab detected by LC–MS affect potency. The collected ^1^H 1D NMR spectra of adalimumab and trastuzumab before and after thermal-pH stress at 40°C also exhibited changes consistent with LC–MS and relative potency measurements indicating that NMR is sensitive to chemical modifications and local structure perturbations in intact mAbs. Moreover, the methyl ^1^H-^13^C 2D NMR spectra of the forced degraded samples for both mAbs remain unchanged relative to the unstressed control confirming that the molecules were not denatured and suggesting that the global HOS is unaffected by the forced degradation conditions. The detected changes in the ^1^H 1D NMR spectra, and ultimately the structure, relative to the unstressed control sample were rapidly assessed using PROFILE and PCA, whereas methyl ^1^H-^13^C 2D NMR spectra were processed using ECHOS.

## Materials and Methods

### Materials

Both adalimumab (Humira, ADL-REF) and trastuzumab (Herceptin, TRA-REF) are therapeutic IgG1 monoclonal antibodies currently licensed and marketed in the US. A total of 84 mg of adalimumab and 84 mg of trastuzumab were purchased, and both were evenly divided into four aliquots. The formulation for adalimumab is 0.8625 mg/mL NaH_2_PO_4_ · 2H_2_O, 1.525 mg/mL Na_2_HPO_4_ · 2H_2_O, 0.3 mg/mL sodium citrate, 1.3 mg/mL citric acid, 6.1625 mg/mL NaCl, 12 mg/mL mannitol, 1 mg/ml polysorbate 80 (PS-80) and pH 5.2. The formulation for trastuzumab is 4.4 mM histidine, 1.7% trehalose, 0.008% PS-20, and pH 6.0.

### Sample Preparation

The sample preparation was identical for adalimumab and trastuzumab, except for the final sample concentrations. Three of the four aliquots for both mAbs were dialyzed into different buffers with varying pH levels. The three pH-stress buffers were 20 mM glutamate (pH 4.5), 20 mM histidine (pH 5.8), and 20 mM tris (pH 7.5). After dialysis, the samples were incubated at 40°C for 4 weeks. The remaining fourth aliquot for each mAb was kept at 4°C during this forced degradation period. All the aliquots were then dialyzed against the same formulation buffer for the respective mAb. Trastuzumab was further concentrated using centrifugal spin columns to a final concentration of ~ 49 mg/mL. Unfortunately, the formulation components for adalimumab were not compatible with the centrifugal spin columns and were therefore left at the initial concentration of about 8 mg/mL. NMR samples were then prepared by adding 10% D_2_O and inserting into a 5 mm Shigemi tube.

### NMR Spectroscopy

The ^1^H NMR spectra were collected with at least three replicates for each sample using the Pulsed Field Gradient Echo (PGSTE) pulse sequence (stebpesgp1s1d) with either 1024 or 2048 scans, a spectral width of 15.6 ppm, an acquisition time of 1.31 s, a diffusion delay of 60 ms, and a diffusion gradient at a strength of 98% for 2 ms [27]. Apodizations of -1.0 Hz exponential and 2.1 Hz Gaussian were applied to the Fourier transformed spectra, which were then phase corrected, baseline corrected, and intensities normalized. The methyl ^1^H-^13^C 2D NMR spectra using the alternate band-SOFAST (ALSOFAST) HMQC (heteronuclear multiple quantum coherence) pulse sequence were collected with 512 scans for adalimumab and 256 scans for trastuzumab, 256 time domain points, an acquisition time of 0.08 s, and spectral widths of 15.6 and 30.0 ppm for ^1^H and ^13^C, respectively [51]. The resulting spectra were then processed using a sine square apodization in both dimensions, zero filling to 4096 points and forward linear prediction to 1024 points in the ^13^C dimension, phase- and baseline-corrected, intensity normalized, and denoised using variable of interest automatic denoising [52].

### Chemometric Analysis

The chemometric analyses were performed using the MBioHOS plugin application for MNova from MestreLab (version 14.0). Blind regions were introduced in regions of the spectra where unsuppressed excipient peaks (polysorbate) or no protein signals were detected to exclude from the analysis. The PROFILE and PCA analyses were performed on both the aliphatic region (0 – 3.2 ppm) and amide/aromatic region (6.0 – 8.8 ppm) of the 1D ^1^H NMR spectra. A broadening factor of 100 Hz was applied for the PROFILE analysis and the correlation coefficient (R value) for the comparison between two spectra were converted to the MNova-defined similarity factor (S) using the following equation:

$$S=10\times \mathrm{log}\left(\frac{R}{1-R}\right)$$

The intra-sample comparisons are between replicate spectra of the same sample, and thus is a measure of the variability in the instrument, which is used as a reference for assessing differences between samples. The inter-sample comparisons are between spectra of different samples and thus is a measure of the variability in the instrument, sample preparation, and protein structure resulting from forced degradation. The statistical limits, mean $\pm$ 2SD and mean $\pm$ 3SD of the intra-sample similarity score, are derived to help evaluate the comparability among different sample groups, where the standard deviation (SD) is estimated as the within-group variance through one way (analysis of variance) ANOVA with the assumption of equal variability in the instrument across groups. The binning for the PCA used bin sizes of 0.01 ppm, which were then normalized to the sum of the bins, center scaled, and bins with a mean value less than 5 were excluded. The shaded regions in the scores plot are the 95% confidence ellipses using the χ^2^ distributions for the given sample set [33]. The Mahalanobis distances were calculated between clusters for each sample, similar to previously published methods [47].

The 2D ^1^H-^13^C methyl NMR spectra were analyzed using the ECHOS method within the MBioHOS plugin for MNova provided by MestreLab. Blind regions were added to eliminate areas of noise and unsuppressed excipient peaks (0.90/13.80 ppm, 1.19/19.09 ppm, 1.32/22.65 ppm, 1.62/24.84 ppm in the ^1^H/^13^C dimension) from the analysis. The contour levels similar to Fig. 3 were used as a threshold for the intensities included in the ECHOS plots (Supplementary Fig. 1). The correlation coefficient (R) of the linear regression for each comparison between the spectra for the control and the forced degraded samples were determined within MBioHOS and are recorded below to assess the differences in the spectra. Lower R values indicate greater differences between the spectra.

## Results

**Fig. 1:**
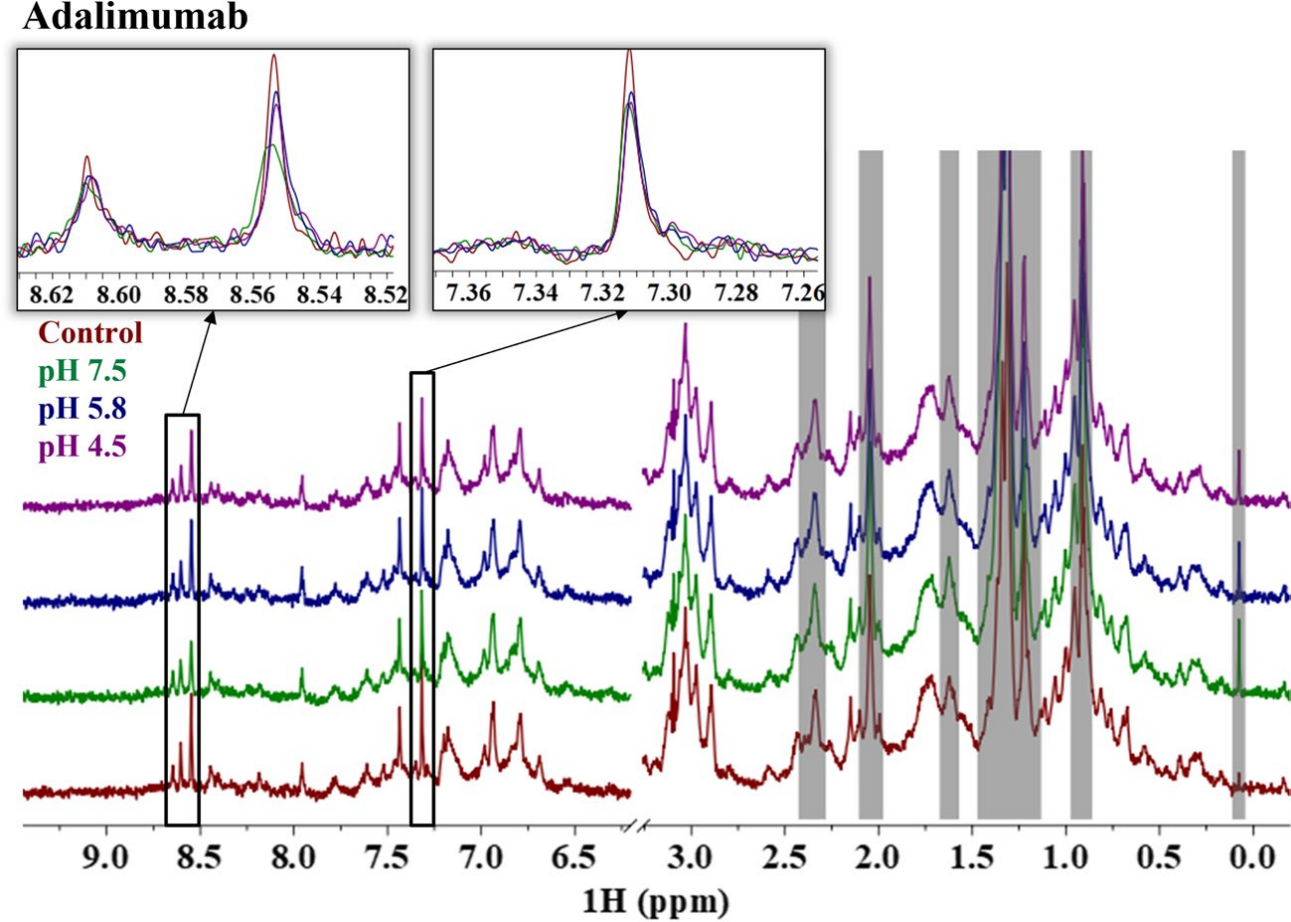
Representative 1D ^1^H NMR spectra of adalimumab for the control sample (red) and the samples stressed at pH 7.5 (green), 5.8 (blue), and 4.5 (purple). Inset figures are meant to highlight regions of interest. The gray bars indicate regions that are excluded from the analysis.

Visual inspection of the representative 1D ^1^H NMR spectra for adalimumab in Fig. 1 shows that the spectra match well to each other regardless of the stress conditions. Additionally, the 2D ^1^H-^13^C HMQC spectra of adalimumab at all conditions also visually match each other well, implying that the overall global structure is maintained (Fig. 3). This is an expected result given that the LC–MS data demonstrates that there are no significant chemical modifications to adalimumab after forced degradation conditions were applied. However, closer inspection of the 1D ^1^H resonances at 7.32, 8.55, and 8.60 ppm show slight differences in intensities from the control spectrum (Fig. 1 insets). The small decrease in intensity at these resonances for the samples subjected to the forced degradation conditions suggests that there are only trace amounts of modifications or subtle conformational changes, which demonstrates the exceptional sensitivity that 1D ^1^H NMR offers to HOS characterization.

**Fig. 2:**
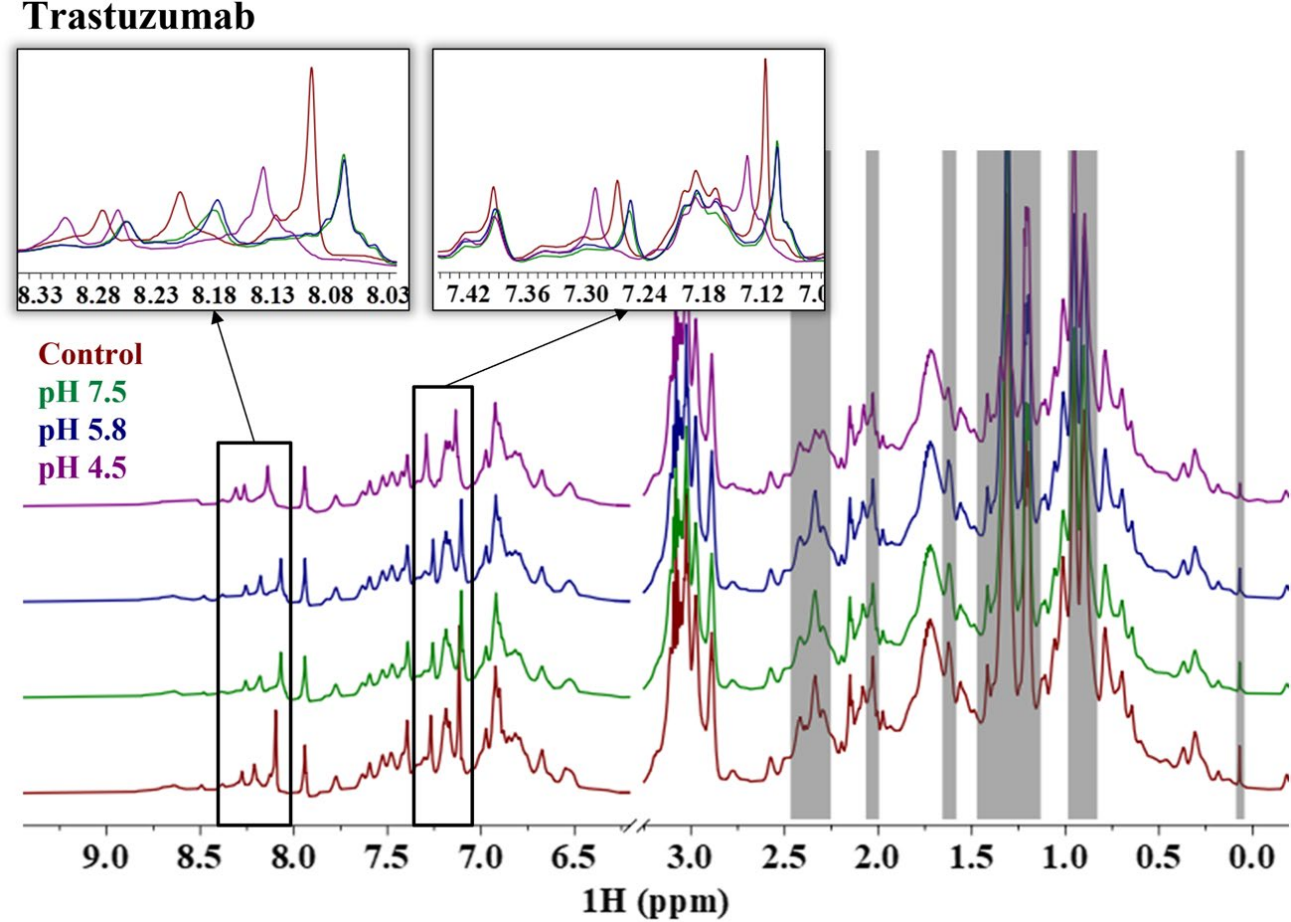
Representative 1D ^1^H NMR spectra of trastuzumab for the control sample (red) and the samples stressed at pH 7.5 (green), 5.8 (blue), and 4.5 (purple). Inset figures are meant to highlight regions of interest. The gray bars indicate regions that are excluded from the analysis.

**Fig. 3:**
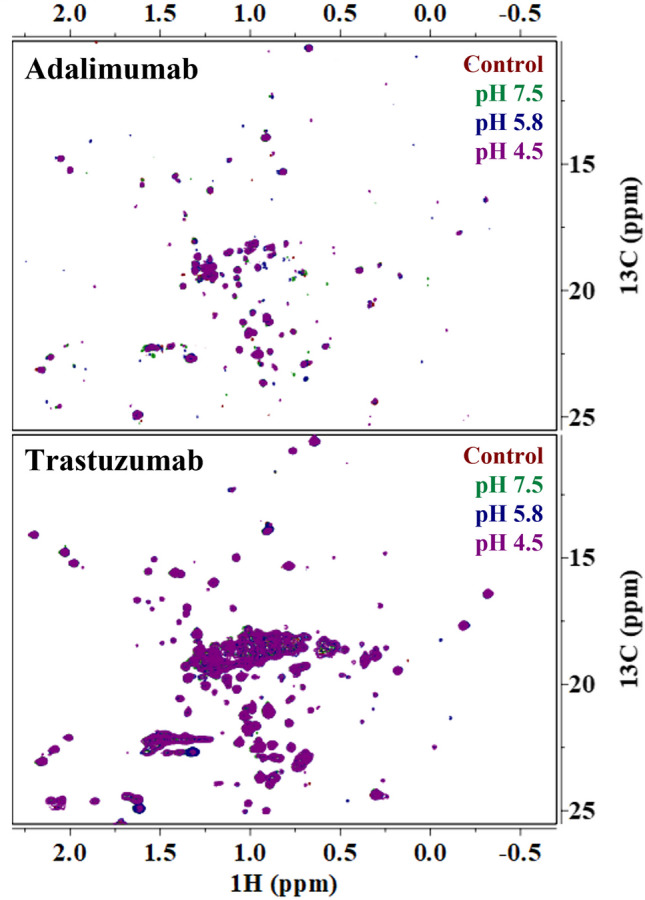
2D ^1^H-^13^C HMQC NMR spectra for the control sample (red) and the samples stressed at pH 7.5 (green), 5.8 (blue), and 4.5 (purple) for both adalimumab (top) and trastuzumab (bottom). The spectra are superimposed in the order of the legend, with the control spectrum as the first layer and the spectrum for the sample stressed at pH 4.5 on top.

Alternatively, while the 2D ^1^H-^13^C NMR spectra of trastuzumab at all conditions match each other well (Fig. 3), the 1D ^1^H spectra show apparent differences around the amide region (8.0–8.4 ppm, 7.0–7.4 ppm), and throughout the aliphatic region (0.0–2.4 ppm) in both chemical shifts and intensities after forced degradation (Fig. 2). This indicates that although the overall global structure is potentially maintained, there are changes to the local structure of trastuzumab, either chemically or conformationally, after thermal-pH forced degradation compared to the unstressed sample (control). The resonances identified in adalimumab showing subtle changes in intensity (7.32, 8.55, and 8.60 ppm) are also perturbed in trastuzumab, but to an even greater extent both in intensity and chemical shift compared to adalimumab (Fig. 2 insets). The forced degradation conditions are causing chemical modifications or local conformational changes to trastuzumab presumably without adversely affecting the overall global protein structure.

For trastuzumab, careful considerations to the context of these differences in the spectra (i.e., extent of changes, spectral region of differences, and structural or functional implications) need to be applied when interpreting the NMR fingerprint and corresponding chemometric results. The resonances at 7.32, 8.55, and 8.60 ppm are of particular interest because they are perturbed in the spectra of the forced degraded samples for both mAbs and the observed chemical shifts are consistent with average amide signals for asparagine (Asn) and aspartic acid (Asp). The average chemical shifts reported in the Biological Magnetic Resonance Data Bank (BMRB) for the Asn/Asp backbone amide proton and Asn sidechain amide protons are 8.3 ± 0.6 and 7.3 ± 0.5 or 7.1 ± 0.5 ppm, respectively [53]. This suggests that the perturbations potentially result from deamidation or isomerization, which is supported by previous LC–MS results measuring high levels of deamidation and isomerization in the CDR regions of trastuzumab. However, further investigation would be required to distinguish the underlying cause of the perturbations observed in the 1D ^1^H spectra. The degree of differences in the spectra is also important to consider because differences in the spectra, however subtle, will be reflected in the chemometric analyses. Subtle changes in the spectrum correspond to smaller conformational changes or changes to a small percent of the ensemble. This is well demonstrated by the contrasting extent of differences in the spectra between adalimumab and trastuzumab and the results of the chemometrics that will be discussed below. While the functional implications are likely dependent on the severity of the structural changes, binding or potency data is necessary to establish causation.

Visual inspection of NMR spectra is quick and a good first step in assessing mAb structure, but there are fundamental shortcomings if further analysis is not performed. A proper visual inspection of mAb spectra still requires an expert in protein NMR and inherently leads to a qualitative assessment. While such an assessment may still have purpose in the drug research and development process, it does not provide adequate metrics for thorough investigations, comparisons, or optimizations. Additionally, it is not always clear to the human eye where differences are occurring or even to what extent in superimposed or stacked spectra. Alternatively, chemometric analysis provides a quantitative assessment with metrics that can be used for detailed structure assessments and can detect differences that even an expert may miss. Beyond the initial visual inspection of the 1D ^1^H NMR spectra above, PCA and PROFILE analysis were performed on the 1D spectra of adalimumab and trastuzumab to further characterize and quantitate the differences between the unstressed and stressed materials. The ECHOS analysis was used to confirm that there are no significant differences in the forced degraded mAb spectra via 2D ^1^H-^13^C NMR relative to the corresponding control.

### ECHOS Analysis of 2D NMR Spectra

The intensities of each spectrum at the contour levels shown in Fig. 3 were measured and plotted against the intensities of the control (unstress) mAb spectrum for both adalimumab and trastuzumab (Supplemental Fig. 1). The correlation coefficient (R value) was then calculated for each plot and recorded in Table I. The R values for the adalimumab spectra between the control and the forced degraded samples are all above 0.95 and for trastuzumab are above 0.97. The difference in the R values is related to the difference in the signal-to-noise of the 2D spectra due to the lower concentration of adalimumab. Considering that there are no benchmark metrics for ECHOS analyses and no differences in the 2D NMR spectra were detected by visual inspection, we consider the R values sufficiently elevated for both mAbs to suggest that the overall global structure is maintained at all conditions. The value in performing this analysis is producing an unbiased correlation coefficient that can be directly used to assess the HOS.

**Table I.** Summary of Chemometric Results

	ECHOS R Values against control		PCA D_M_ from control	
Stress Condition	Adalimumab	Trastuzumab	Adalimumab	Trastuzumab
pH 7.5	0.9501	0.9826	3.74	40.1
pH 5.8	0.9505	0.9752	4.97	11.1
pH 4.5	0.9558	0.9756	5.16	57.5

The R values (correlation coefficient) of the linear regression from each ECHOS analysis plot in Supplemental Fig. 1 are recorded (left side of table) for adalimumab and trastuzumab for the control *versus* the samples forced degraded at pH 7.5, 5.8, and 4.5. Mahalanobis distances from the control for each stress condition for both adalimumab and trastuzumab are recorded on the right side of the table

### PCA of 1D ^1^H NMR Spectra of Adalimumab and Trastuzumab

The MBioHOS plugin to MNova provided by MestreLab was used to perform the PCA. The same parameters were used across adalimumab and trastuzumab, which were optimized for performance to maximize the explained variance and minimize residuals of the first two principal components. Key parameters that were optimized (values used are in parentheses) include the bin sizes (0.01 ppm), filtering method (mean filtering), and scaling method (center scaling). Normalization did not have a significant effect on the analysis given that the concentrations were very similar and different normalization methods had similar effects. Datasets with greater variation in concentration between samples may experience an added benefit to optimizing the normalization scheme. The tradeoff in the bin dimensions is between higher resolution and computing time, which is much more of a consideration in 2D spectra than in 1D spectra. We tested different bin dimensions in the MBioHOS plugin and found that 0.01 ppm for 1D NMR spectra gave appropriate resolution while still completing the calculation within seconds. Filtering the bins is a way to reduce contributions from the noise and other low signal-to-noise features (i.e., artifacts and impurities). Although different methods gave acceptable results, we used a 5.0 mean filter, which eliminated bins with a mean intensity value below 5. Lastly, the scaling method had the greatest impact on the performance of the PCA and often depended on other processing parameters (i.e., normalization and filtering). Although many different scaling schemes exist, such as centering, autoscaling, range, vast, pareto, and level, selection depends on the characteristics of the data and the interest of the analysis [54]. Given that most of the spectra are comparable, and we are interested in extracting differences in the data, center scaling was selected. Additionally, blind regions (gray regions in Figs. 1 and 2) were also applied to reduce the contributions from the noise and unsuppressed excipient peaks, mainly from polysorbate, to the calculation by eliminating them from the analysis. This biases the analysis towards differences in the fingerprint of the protein and minimizes contributions from the noise and excipients.

**Fig. 4:**
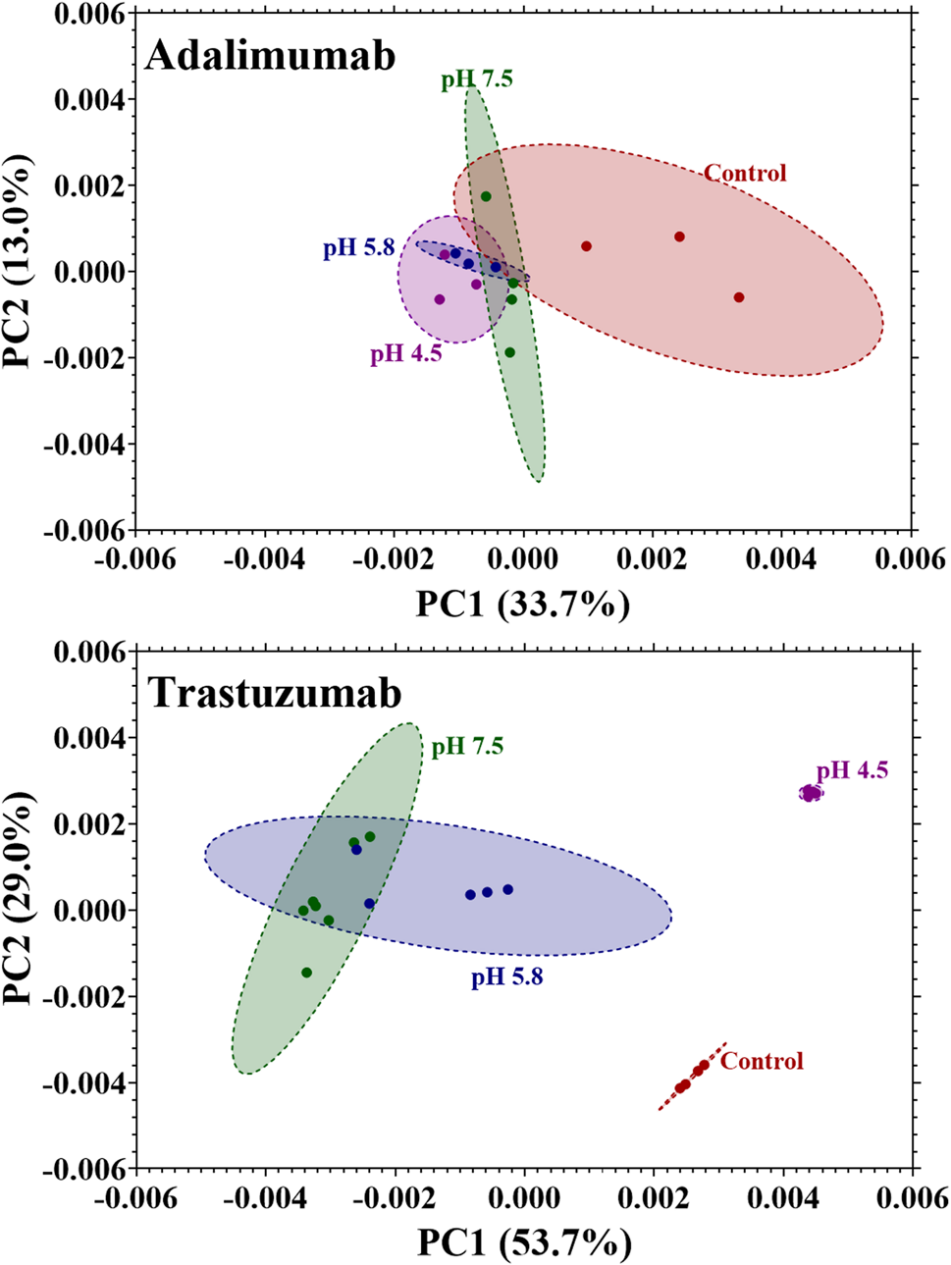
PCA scores plot of PC1 and PC2 of adalimumab (top) and trastuzumab (bottom) samples. The control samples (red) and the samples stressed at pH 7.5 (green), 5.8 (blue), and 4.5 (purple) are represented as points and the 95% confidence interval as ellipses.

The score plots for the first two principal components of the PCA for the 1D NMR spectra of adalimumab and trastuzumab are shown in Fig. 4. The distances between clusters are shorter in the PCA for adalimumab than they are for trastuzumab, which demonstrates that the differences in trastuzumab are more significant than those observed in adalimumab. The calculated Mahalanobis distances (D_M_) between clusters for the stressed samples to the control sample are shown in Table I, which quantitates these distances between clusters in the score plots by considering the center of the clusters and the size and orientation of the 95% confidence ellipses. The corresponding distances of the stressed samples to the control cluster are about 2–11 times longer for trastuzumab than for adalimumab, further demonstrating that the differences in trastuzumab are much more significant than in adalimumab. The orientation of the clusters in relation to the principal components also provides meaningful information regarding sample characteristics. For instance, the clusters along PC1 in the score plot for adalimumab show little overlap between the control and the stress samples, whereas along PC2 there is less variation.

Components of the loadings matrix (weights or coefficients from which the principal components are defined) for adalimumab show that none of the first four PCs are dominated by any region of the spectrum, including the amide region or peaks consistent with Asn and Asp. This suggests that there are no significant differences in the spectra of adalimumab that greatly influence the PCs and that any differences detected by visual inspection are subtle and dispersed. Neither PC1 nor PC2 have a strong influence from differences in the amide region and so any differences in the scores plot does not reflect changes in that region. The loadings matrix for trastuzumab shows that the first two components are dominated by the bin intensities in the amide region, specifically those corresponding to peaks consistent with Asn and Asp. This means that the differences in the intensity and chemical shift of the amide region detected by visual inspection influence both PC1 and PC2. The variation in scores along PC1 and PC2 inform on differences detected in the amide region. The score plot for trastuzumab shows no overlapping of the clusters between the control and the stressed samples along both PC1 and PC2, which indicates that there are major differences in the amide region between the control and stressed samples. When considering only the stressed samples, there is less variation along PC2 than there is along PC1. This suggests that although there are differences among the stressed samples in the amide region, there may still be some similarities. The greatest overlap of clusters in the score plot for trastuzumab is between the samples stressed at pH 7.5 and 5.8 and suggests that the mechanism for degradation at these conditions is similar, while also being distinct from the degradation mechanism at pH 4.5. Further investigation is required to confirm this observation, but it is important to note that simple 1D NMR fingerprinting is capable of detecting these nuances in the forced degradation experiment. These observations are consistent with the visual inspection of the spectra where differences in the spectra of adalimumab are not as apparent as those in trastuzumab.

### PROFILE analysis of 1D ^1^H NMR Spectra of Adalimumab and Trastuzumab

**Fig. 5:**
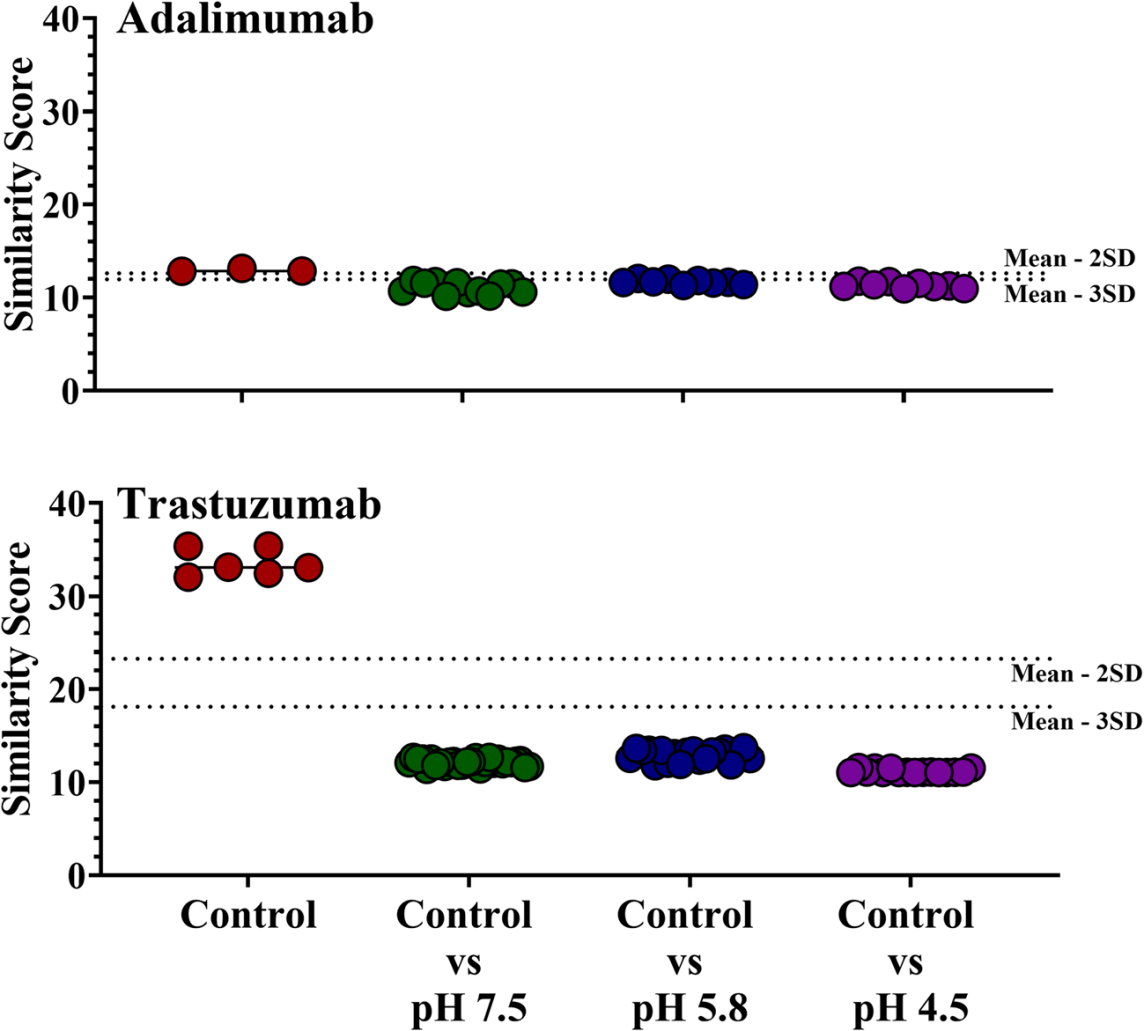
Summary of PROFILE analysis results of adalimumab (top) and trastuzumab (bottom) samples. The dotted lines represent 2SD and 3SD from the mean value of the control intra-sample comparisons. Results for the control are shown in red, pH 7.5 in green, pH 5.8 in blue, and pH 4.5 in purple.

The PROFILE analysis was another chemometric tool used to quantitate differences in the spectra of the forced degradation samples, which can be implemented to confirm the results from the PCA or be used as a complementary technique. This study used the MBioHOS plugin to perform the PROFILE analysis with a broadening factor optimized to a value of 100 Hz, which provided good resolution while keeping the computation time within seconds. The results of the PROFILE analysis are summarized in Fig. 5 where each point represents a pairwise comparison with a given similarity score calculated in MBioHOS. The first group of points for both mAbs are the intra-sample comparisons for the control, or in other words the comparisons between replicate spectra of the control. The equivalent calculations were performed for the stressed samples with similar results but are not shown in the figure for the purpose of simplicity since the assumption is that the intra-sample comparisons represent variation in the instrument. With this assumption, the standard deviation (SD) was calculated using the within-group variance of a one-way ANOVA analysis of all the intra-sample comparisons. The dotted lines in the plots are 2SD and 3SD from the mean of the control intra-sample comparisons. Points within 2SD from the mean are considered matching spectra, between 2 and 3SD from the mean spectra have slight differences, and beyond 3SD of the mean spectra have significant differences. These lines are meant to help benchmark the differences in similarity scores between the intra-sample comparisons of the control with the inter-sample comparisons between the stressed samples and the control. The following groups of points are the inter-sample comparisons between the control and each of the stressed samples with the solid bar representing the mean value.

The similarity scores for adalimumab are lower in value than those for trastuzumab. The mean value for the intra-sample comparisons of the control is 12.9 for adalimumab and 31.3 for trastuzumab. This is a result of the lower signal-to-noise of the adalimumab spectra compared to that of trastuzumab, which demonstrates the sensitivity of PROFILE to the noise of the spectrum. Studies that implement PROFILE should seek to maximize the signal-to-noise of their spectra. The PROFILE plot for adalimumab shows that although most of the inter-sample comparison points are beyond 3SD from the mean, there are still a few peaks within this threshold. In contrast, the inter-sample comparisons for trastuzumab are further beyond 3SD of the mean, by about 5.79 – 6.38 points from the mean, compared to adalimumab, where the mean values for inter-sample comparisons are only 0.28 – 0.91 points beyond the 3SD of the mean. These observations are consistent with the visual inspection and PCA results in that there are only subtle differences in adalimumab, while trastuzumab has much more noticeable differences in the spectra. The PROFILE analysis in this study demonstrates the sensitivity in detecting differences after forced degradation conditions, even when these differences are below the detection limit of other techniques.

## Discussion

The advantage to the PROFILE analysis is that it does not require as many replicate spectra (or lots) as PCA since it is a pairwise comparison, although the analysis is strengthened with more replicate spectra. In this study, we compared the same spectra as the PCA to demonstrate the utility and complementarity of the PROFILE analysis. However, if only a few representative lots can be secured or experiment times need to be short, i.e., to accommodate the instability of the sample or because spectrometer time is limited, the PROFILE analysis is an acceptable alternative to the more intensive PCA. It should be noted that the PROFILE analysis is more sensitive to differences in the 1D spectra, including noise, which should be carefully considered when interpreting the results. Additionally, assuming that the processing of the NMR data is the same, there are fewer parameters to optimize for PROFILE (broadening factor) compared to PCA (binning scheme, normalization, filtering, and scaling). This lends itself to easier analyses and better transferability of the method. The advantage of using PCA is the multidimensionality of the results. Even when only considering the first two principal components, the second dimension (PC2) provides added resolution of the differences within and between clusters that enhances HOS characterization. Inherently, the first two principal components account for the most variance in the data, but other studies may find value in considering additional principal components. Our study shows that both PCA and PROFILE have value in assessing HOS comparability, and the complementarity between these methods provides a strong analysis when both are applied.

## Conclusions

We demonstrated a new application of established NMR chemometric tools (ECHOS, PROFILE, and PCA) in a case study involving forced degradation of adalimumab (Humira, ADL-REF) and trastuzumab (Herceptin, TRA-REF). We used a commercially available software package to perform these calculations and described the necessary considerations for optimal results. Our study was able to detect large chemical shift and intensity differences in the spectra of trastuzumab, consistent with LC–MS data, and was sensitive enough to detect subtle differences in intensity at key regions of the spectra for adalimumab. Cross-reference with the BMRB indicates that these regions of greatest differences in the spectra are consistent with amide proton resonances for the backbone and Asn sidechain, which suggests that the stressed samples in this study have Asn deamidation and Asp isomerization products. Based on the 2D NMR data and ECHOS analyses, we assume that despite these chemical modifications, the global structures of adalimumab and trastuzumab are unperturbed. PCA of the NMR spectra captures and quantitates the subtle differences in adalimumab and the larger differences in trastuzumab. Additionally, the PCA score plots provide additional insight to the degradation mechanism between the different forced degradation conditions. The PROFILE analysis provides a complementary and quantitative measure of the differences between the control and the stressed samples for the mAbs in this study, which are consistent with the visual inspection and PCA results. Although our focus in this publication centered on forced degradation samples, the principles outlined can be translated to other comparative studies in process and product development (i.e., analytical comparability), as well as biosimilarity exercises.

### Supplementary Information

Below is the link to the electronic supplementary material.

Supplementary file1 (DOCX 327 KB)
