# Supplementary material for: Application of NMR and Chemometric Analyses to Better Understand the Quality Attributes in pH and Thermally Degraded Monoclonal Antibodies
Source: Pharm Res. 2023 Oct 5;40(10):2457–67. doi: 10.1007/s11095-023-03600-2 (PMC10661726; doi:10.1007/s11095-023-03600-2)
Supplement: Supplementary file 1 — Supplementary file1 (DOCX 327 KB) [file 11095_2023_3600_MOESM1_ESM.docx]

**SUPPLEMENTAL INFORMATION**

**
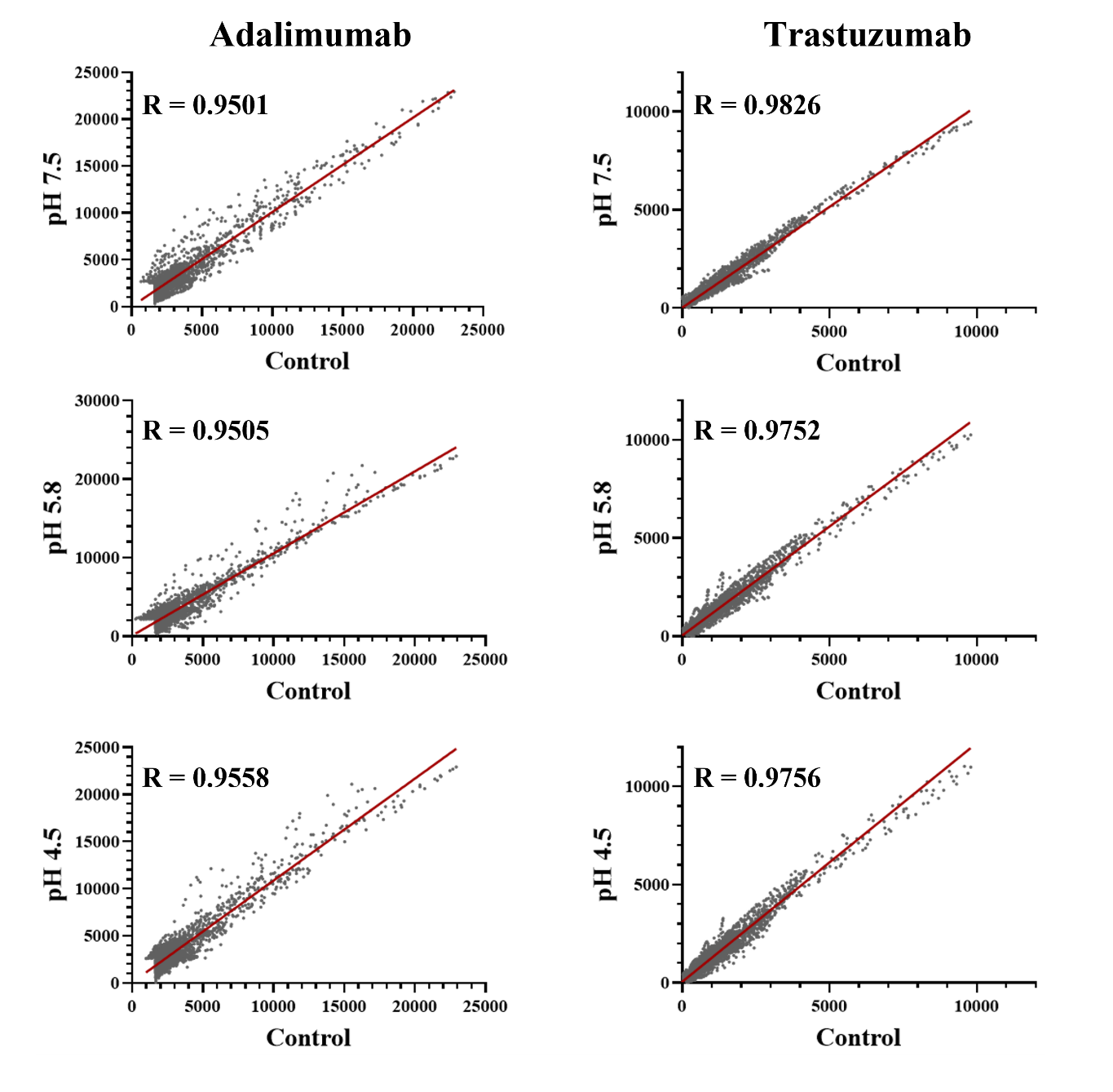
**

**Figure S1: ECHOS plots of control spectrum intensities versus the spectra intensity of forced degraded samples for adalimumab (left) and trastuzumab (right).** The ECHOS plots for the control and the forced degraded sample at pH 7.5 (top), pH 5.8 (middle), and pH 4.5 (bottom) are shown where the gray points are the intensity cross correlation points, and the red line is the line of best fit. The R values recorded are the measure of goodness of fit calculated by MBioHOS for each corresponding plot.
